# 1-Methyl-3-(4-vinyl­benz­yl)imidazolium hexa­fluoro­phosphate

**DOI:** 10.1107/S1600536810000437

**Published:** 2010-01-16

**Authors:** Xiang-Yong Lu, Jia-Feng Sun, Lin Zhang, Xue-Tai Chen

**Affiliations:** a State Key Laboratory of Coordination Chemistry, School of Chemistry and Chemical Engineering, Nanjing University, Nanjing 210093, People’s Republic of China; b Department of Materials and Chemical Engineering, Taishan University, Taian 271021, People’s Republic of China; c Research Centre of Laser Fusion, CAEP, Mianyang 621900, People’s Republic of China

## Abstract

In the title compound, C_13_H_15_N_2_^+^·PF_6_^−^, the dihedral angle between the two aromatic rings is 85.48 (7)°. In the crystal, C—H⋯F hydrogen bonds connect the imidazolium and hexa­fluoro­phosphate ions.

## Related literature

For *N*-heterocyclic carbenes, see: Herrmann (2002 ▶). For the synthesis of the title compound, see: Kim *et al.* (2005 ▶). For a silver compound with 1-methyl-3-(4-vinyl­benz­yl)imidazol-2-yl­idene, see: Lu *et al.* (2009 ▶).
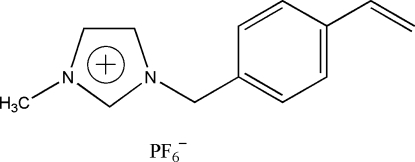


## Experimental

### 

#### Crystal data

C_13_H_15_N_2_^+^·PF_6_^−^
*M* *_r_* = 344.24
Orthorhombic, 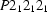

*a* = 10.482 (2) Å
*b* = 11.272 (3) Å
*c* = 12.556 (3) Å
*V* = 1483.4 (6) Å^3^
*Z* = 4
Mo *K*α radiation
μ = 0.25 mm^−1^
*T* = 298 K
0.32 × 0.29 × 0.26 mm

#### Data collection

Bruker SMART CCD area-detector diffractometer
Absorption correction: multi-scan (*SADABS*; Bruker, 2005 ▶) *T*_min_ = 0.925, *T*_max_ = 0.939
9185 measured reflections
3542 independent reflections
3407 reflections with *I* > 2σ(*I*)
*R*_int_ = 0.085

#### Refinement

*R*[*F* ^2^ > 2σ(*F* ^2^)] = 0.047
*wR*(*F* ^2^) = 0.127
*S* = 1.03
3542 reflections
200 parameters
H-atom parameters constrained
Δρ_max_ = 0.65 e Å^−3^
Δρ_min_ = −0.89 e Å^−3^
Absolute structure: Flack (1983 ▶), 1484 Friedel pairs
Flack parameter: 0.05 (13)

### 

Data collection: *SMART* (Bruker, 2005 ▶); cell refinement: *SAINT* (Bruker, 2005 ▶); data reduction: *SAINT*; program(s) used to solve structure: *SHELXS97* (Sheldrick, 2008 ▶); program(s) used to refine structure: *SHELXL97* (Sheldrick, 2008 ▶); molecular graphics: *XP* in *SHELXTL* (Sheldrick, 2008 ▶); software used to prepare material for publication: *SHELXL97*.

## Supplementary Material

Crystal structure: contains datablocks I, global. DOI: 10.1107/S1600536810000437/bt5162sup1.cif

Structure factors: contains datablocks I. DOI: 10.1107/S1600536810000437/bt5162Isup2.hkl

Additional supplementary materials:  crystallographic information; 3D view; checkCIF report

## Figures and Tables

**Table 1:** Hydrogen-bond geometry (Å, °)

*D*—H⋯*A*	*D*—H	H⋯*A*	*D*⋯*A*	*D*—H⋯*A*
C4—H4⋯F1^i^	0.93	2.36	3.266 (3)	164
C3—H3⋯F6^ii^	0.93	2.25	3.044 (3)	143

Symmetry codes: (i) 
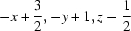
; (ii) 
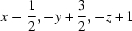
.

**Fig. 1.**
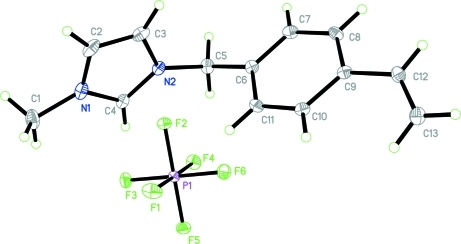
The molecular structure of title compound with atom labels and 30% probability displacement ellipsoids for non-H atoms.

**Fig. 2.**
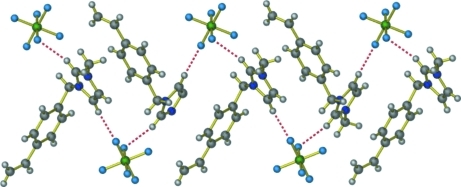
A packing diagram of the title compound. Hydrogen bonds are shown as dashed lines.

## supplementary crystallographic information

### Comment

N-heterocyclic carbenes have attracted great attention as they can act as efficient supporting ligands in organometallic chemistry and homogeneous catalysis (Herrmann, 2002). Imidazolium salts are the useful precusors of the N-heterocyclic carbenes. The silver compound  with 1-methyl-3-(4-vinylbenzyl)imidazol-2-ylidene has been synthesized (Lu *et al.*, 2009).

The structure of the title compound is shown in Fig. 1. The two aromatic rings enclose  a dihedral angle of 85.48 (7)°. The hexafluorophosphate anions are linked to 1-methyl-3-(4-vinylbenzyl)imidazolium cations *via* C—H···F hydrogen bonds (Table 1 and Fig. 2). These interacions stabilize the crystal structure.

### Experimental

1-Methyl-3-(4-vinylbenzyl)imidazolium chloride was synthesized according to the literature method (Kim *et al.*, 2005). The resulting white solid (0.70 g, 3.00 mmol) was dissolved in acetone (30 ml) and then potassium hexafluorophosphate (1.38 g, 7.50 mmol) was added. The mixture was stirred at room temperature for 26 h and the solvent was removed under reduced pressure. The residue was then dissolved in distilled water and extracted with CH_2_Cl_2_. The white solid was abtained after the removal of solvent. Yield: 0.78 g (76%). Anal. Calcd for C_13_H_15_F_6_N_2_P: C,45.36; H, 4.39; N, 8.14. Found: C, 45.13; H, 4.03; N, 8.35. The elemental analyses were performed with Vario MICRO elemental analyzer.

### Refinement

The H-atoms were included in the riding-model approximation with C—H = 0.93 Å and C—H = 0.96 Å, and with *U*_iso_(H) = 1.2*U*_eq_(C-aromatic) and *U*_iso_(H) = 1.5*U*_eq_(*C*-methyl).

### Crystal data

**Table:**

C_13_H_15_N_2_^+^·PF_6_^−^	*F*(000) = 704
*M**_r_* = 344.24	*D*_x_ = 1.541 Mg m^−^^3^
Orthorhombic, *P*2_1_2_1_2_1_	Mo *K*α radiation, λ = 0.71073 Å
Hall symbol: P 2ac 2ab	Cell parameters from 6513 reflections
*a* = 10.482 (2) Å	θ = 2.4–28.3°
*b* = 11.272 (3) Å	µ = 0.25 mm^−^^1^
*c* = 12.556 (3) Å	*T* = 298 K
*V* = 1483.4 (6) Å^3^	Block, colorless
*Z* = 4	0.32 × 0.29 × 0.26 mm

### Data collection

**Table:**

Bruker SMART CCD area-detector diffractometer	3542 independent reflections
Radiation source: fine-focus sealed tube	3407 reflections with *I* > 2σ(*I*)
graphite	*R*_int_ = 0.085
phi and ω scans	θ_max_ = 28.3°, θ_min_ = 2.4°
Absorption correction: multi-scan (*SADABS*; Bruker, 2005)	*h* = −13→11
*T*_min_ = 0.925, *T*_max_ = 0.939	*k* = −14→14
9185 measured reflections	*l* = −16→14

### Refinement

**Table:**

Refinement on *F*^2^	Secondary atom site location: difference Fourier map
Least-squares matrix: full	Hydrogen site location: inferred from neighbouring sites
*R*[*F*^2^ > 2σ(*F*^2^)] = 0.047	H-atom parameters constrained
*wR*(*F*^2^) = 0.127	*w* = 1/[σ^2^(*F*_o_^2^) + (0.0549*P*)^2^ + 1.5672*P*] where *P* = (*F*_o_^2^ + 2*F*_c_^2^)/3
*S* = 1.03	(Δ/σ)_max_ < 0.001
3542 reflections	Δρ_max_ = 0.65 e Å^−^^3^
200 parameters	Δρ_min_ = −0.89 e Å^−^^3^
0 restraints	Absolute structure: Flack (1983), 1484 Friedel pairs
Primary atom site location: structure-invariant direct methods	Flack parameter: 0.05 (13)

### Special details

**Table:**

Geometry. All e.s.d.'s (except the e.s.d. in the dihedral angle between two l.s. planes) are estimated using the full covariance matrix. The cell e.s.d.'s are taken into account individually in the estimation of e.s.d.'s in distances, angles and torsion angles; correlations between e.s.d.'s in cell parameters are only used when they are defined by crystal symmetry. An approximate (isotropic) treatment of cell e.s.d.'s is used for estimating e.s.d.'s involving l.s. planes.
Refinement. Refinement of *F*^2^ against ALL reflections. The weighted *R*-factor *wR* and goodness of fit *S* are based on *F*^2^, conventional *R*-factors *R* are based on *F*, with *F* set to zero for negative *F*^2^. The threshold expression of *F*^2^ > σ(*F*^2^) is used only for calculating *R*-factors(gt) *etc*. and is not relevant to the choice of reflections for refinement. *R*-factors based on *F*^2^ are statistically about twice as large as those based on *F*, and *R*- factors based on ALL data will be even larger.

### Fractional atomic coordinates and isotropic or equivalent isotropic displacement parameters (Å^2^)

**Table:**

	*x*	*y*	*z*	*U*_iso_*/*U*_eq_	
C1	0.8710 (3)	0.2998 (3)	0.0944 (2)	0.0351 (7)	
H1A	0.9248	0.2527	0.1396	0.053*	
H1B	0.9017	0.3800	0.0931	0.053*	
H1C	0.8722	0.2679	0.0235	0.053*	
C2	0.6394 (3)	0.3609 (2)	0.0958 (2)	0.0283 (6)	
H2	0.6415	0.4128	0.0382	0.034*	
C3	0.5369 (3)	0.3342 (2)	0.1548 (2)	0.0249 (5)	
H3	0.4549	0.3641	0.1460	0.030*	
C4	0.6999 (3)	0.2335 (2)	0.21741 (19)	0.0204 (5)	
H4	0.7497	0.1826	0.2583	0.025*	
C5	0.4948 (3)	0.2031 (3)	0.3150 (2)	0.0228 (5)	
H5A	0.4678	0.2657	0.3630	0.027*	
H5B	0.5440	0.1461	0.3558	0.027*	
C6	0.3787 (2)	0.1426 (2)	0.26935 (19)	0.0192 (5)	
C7	0.2587 (3)	0.1657 (2)	0.3101 (2)	0.0236 (5)	
H7	0.2489	0.2225	0.3632	0.028*	
C8	0.1529 (3)	0.1050 (3)	0.2724 (2)	0.0234 (5)	
H8	0.0729	0.1216	0.3008	0.028*	
C9	0.1643 (2)	0.0194 (2)	0.19242 (19)	0.0191 (5)	
C10	0.2861 (2)	−0.0017 (2)	0.14997 (18)	0.0190 (5)	
H10	0.2960	−0.0569	0.0956	0.023*	
C11	0.3914 (2)	0.0584 (2)	0.1878 (2)	0.0199 (5)	
H11	0.4714	0.0429	0.1590	0.024*	
C12	0.0501 (3)	−0.0449 (2)	0.1563 (2)	0.0230 (5)	
H12	−0.0282	−0.0177	0.1812	0.028*	
C13	0.0480 (3)	−0.1377 (3)	0.0917 (2)	0.0260 (5)	
H13A	0.1239	−0.1681	0.0647	0.031*	
H13B	−0.0294	−0.1725	0.0733	0.031*	
F1	0.67298 (19)	0.98396 (17)	0.85265 (12)	0.0364 (4)	
F2	0.75104 (15)	0.85036 (13)	0.96990 (13)	0.0245 (3)	
F3	0.54994 (15)	0.92291 (15)	0.99001 (14)	0.0297 (4)	
F4	0.70480 (17)	0.97546 (17)	1.10592 (12)	0.0305 (4)	
F5	0.62797 (17)	1.11050 (14)	0.98847 (14)	0.0309 (4)	
F6	0.82848 (16)	1.03763 (16)	0.96874 (17)	0.0359 (4)	
N1	0.7402 (2)	0.2980 (2)	0.13540 (17)	0.0247 (5)	
N2	0.5760 (2)	0.25420 (19)	0.23092 (17)	0.0191 (4)	
P1	0.68904 (6)	0.98115 (6)	0.97936 (5)	0.01785 (15)	

### Atomic displacement parameters (Å^2^)

**Table:**

	*U*^11^	*U*^22^	*U*^33^	*U*^12^	*U*^13^	*U*^23^
C1	0.0343 (15)	0.0449 (17)	0.0259 (13)	−0.0109 (14)	0.0098 (12)	−0.0043 (13)
C2	0.0468 (16)	0.0210 (12)	0.0170 (11)	−0.0073 (12)	−0.0057 (12)	0.0022 (10)
C3	0.0371 (14)	0.0197 (12)	0.0179 (11)	0.0005 (11)	−0.0092 (11)	0.0012 (9)
C4	0.0257 (12)	0.0206 (12)	0.0149 (10)	−0.0049 (10)	−0.0021 (9)	−0.0001 (8)
C5	0.0254 (12)	0.0298 (13)	0.0130 (10)	−0.0031 (11)	−0.0006 (10)	0.0008 (10)
C6	0.0227 (11)	0.0222 (12)	0.0128 (10)	0.0002 (10)	−0.0020 (9)	0.0007 (9)
C7	0.0295 (13)	0.0235 (12)	0.0180 (10)	0.0022 (11)	0.0003 (10)	−0.0052 (10)
C8	0.0218 (12)	0.0274 (13)	0.0212 (11)	0.0045 (10)	0.0049 (10)	−0.0042 (10)
C9	0.0244 (12)	0.0188 (11)	0.0142 (9)	0.0014 (9)	−0.0009 (9)	0.0024 (9)
C10	0.0266 (12)	0.0191 (12)	0.0114 (9)	0.0024 (9)	0.0013 (9)	−0.0005 (8)
C11	0.0197 (11)	0.0246 (12)	0.0154 (10)	0.0046 (10)	0.0016 (9)	0.0024 (9)
C12	0.0210 (11)	0.0288 (13)	0.0192 (11)	−0.0004 (10)	0.0015 (10)	0.0016 (10)
C13	0.0262 (12)	0.0297 (14)	0.0223 (12)	−0.0030 (11)	−0.0030 (10)	−0.0017 (11)
F1	0.0519 (11)	0.0436 (10)	0.0136 (7)	0.0145 (9)	−0.0002 (7)	−0.0030 (7)
F2	0.0282 (8)	0.0221 (7)	0.0234 (7)	0.0037 (6)	−0.0019 (7)	−0.0001 (6)
F3	0.0214 (7)	0.0343 (9)	0.0336 (9)	−0.0042 (6)	−0.0004 (7)	0.0058 (7)
F4	0.0384 (9)	0.0382 (9)	0.0149 (7)	0.0029 (8)	0.0031 (6)	0.0037 (6)
F5	0.0343 (8)	0.0240 (8)	0.0343 (9)	0.0064 (7)	0.0045 (8)	0.0040 (7)
F6	0.0238 (8)	0.0317 (9)	0.0521 (11)	−0.0055 (7)	−0.0052 (8)	−0.0098 (8)
N1	0.0333 (12)	0.0256 (11)	0.0153 (9)	−0.0103 (10)	0.0007 (9)	−0.0018 (9)
N2	0.0234 (10)	0.0191 (10)	0.0147 (9)	−0.0025 (8)	−0.0026 (8)	0.0011 (8)
P1	0.0191 (3)	0.0204 (3)	0.0141 (3)	0.0009 (2)	−0.0008 (2)	0.0002 (2)

### Geometric parameters (Å, °)

**Table:**

C1—N1	1.464 (4)	C7—H7	0.9300
C1—H1A	0.9600	C8—C9	1.398 (4)
C1—H1B	0.9600	C8—H8	0.9300
C1—H1C	0.9600	C9—C10	1.404 (3)
C2—C3	1.339 (4)	C9—C12	1.471 (4)
C2—N1	1.367 (4)	C10—C11	1.379 (4)
C2—H2	0.9300	C10—H10	0.9300
C3—N2	1.377 (3)	C11—H11	0.9300
C3—H3	0.9300	C12—C13	1.324 (4)
C4—N1	1.330 (3)	C12—H12	0.9300
C4—N2	1.330 (3)	C13—H13A	0.9300
C4—H4	0.9300	C13—H13B	0.9300
C5—N2	1.473 (3)	F1—P1	1.6001 (17)
C5—C6	1.508 (4)	F2—P1	1.6156 (16)
C5—H5A	0.9700	F3—P1	1.6046 (17)
C5—H5B	0.9700	F4—P1	1.5988 (16)
C6—C7	1.383 (4)	F5—P1	1.5964 (17)
C6—C11	1.403 (4)	F6—P1	1.5998 (18)
C7—C8	1.387 (4)		
			
N1—C1—H1A	109.5	C11—C10—C9	120.9 (2)
N1—C1—H1B	109.5	C11—C10—H10	119.5
H1A—C1—H1B	109.5	C9—C10—H10	119.5
N1—C1—H1C	109.5	C10—C11—C6	120.5 (2)
H1A—C1—H1C	109.5	C10—C11—H11	119.7
H1B—C1—H1C	109.5	C6—C11—H11	119.7
C3—C2—N1	107.6 (2)	C13—C12—C9	126.3 (3)
C3—C2—H2	126.2	C13—C12—H12	116.9
N1—C2—H2	126.2	C9—C12—H12	116.9
C2—C3—N2	107.0 (3)	C12—C13—H13A	120.0
C2—C3—H3	126.5	C12—C13—H13B	120.0
N2—C3—H3	126.5	H13A—C13—H13B	120.0
N1—C4—N2	108.3 (2)	C4—N1—C2	108.6 (2)
N1—C4—H4	125.9	C4—N1—C1	125.3 (3)
N2—C4—H4	125.9	C2—N1—C1	126.0 (3)
N2—C5—C6	111.8 (2)	C4—N2—C3	108.5 (2)
N2—C5—H5A	109.3	C4—N2—C5	125.9 (2)
C6—C5—H5A	109.3	C3—N2—C5	125.6 (2)
N2—C5—H5B	109.3	F5—P1—F4	90.40 (10)
C6—C5—H5B	109.3	F5—P1—F6	90.51 (10)
H5A—C5—H5B	107.9	F4—P1—F6	90.25 (10)
C7—C6—C11	118.9 (2)	F5—P1—F1	90.63 (10)
C7—C6—C5	120.6 (2)	F4—P1—F1	178.83 (11)
C11—C6—C5	120.5 (2)	F6—P1—F1	90.30 (11)
C6—C7—C8	120.6 (2)	F5—P1—F3	90.19 (10)
C6—C7—H7	119.7	F4—P1—F3	89.69 (10)
C8—C7—H7	119.7	F6—P1—F3	179.30 (10)
C7—C8—C9	121.2 (2)	F1—P1—F3	89.74 (10)
C7—C8—H8	119.4	F5—P1—F2	179.86 (11)
C9—C8—H8	119.4	F4—P1—F2	89.71 (9)
C8—C9—C10	117.8 (2)	F6—P1—F2	89.39 (9)
C8—C9—C12	119.5 (2)	F1—P1—F2	89.27 (9)
C10—C9—C12	122.7 (2)	F3—P1—F2	89.91 (9)

### Hydrogen-bond geometry (Å, °)

**Table:**

*D*—H···*A*	*D*—H	H···*A*	*D*···*A*	*D*—H···*A*
C4—H4···F1^i^	0.93	2.36	3.266 (3)	164
C3—H3···F6^ii^	0.93	2.25	3.044 (3)	143

Symmetry codes: (i) −*x*+3/2, −*y*+1, *z*−1/2; (ii) *x*−1/2, −*y*+3/2, −*z*+1.

## References

[bb1] Bruker (2005). *SMART*, *SAINT*, *SADABS* Bruker AXS Inc., Madison, Wisconsin, USA.

[bb2] Flack, H. D. (1983). *Acta Cryst.* A**39**, 876–881.

[bb3] Herrmann, W. A. (2002). *Angew. Chem. Int. Ed. Engl.* **41**, 1290–1309.

[bb4] Kim, J.-H., Kim, J.-W., Shokouhimehr, M. & Lee, Y.-S. (2005). *J. Org. Chem.* **70**, 6714–6720. 10.1021/jo050721m 16095291

[bb5] Lu, X.-Y., CHen, F., Xu, W.-F. & Chen, X.-T. (2009). *Inorg. Chim. Acta*, **362**, 5113–5116.

[bb6] Sheldrick, G. M. (2008). *Acta Cryst.* A**64**, 112–122. 10.1107/S0108767307043930 18156677

